# Validation of plasma microRNAs as biomarkers for myotonic dystrophy type 1

**DOI:** 10.1038/srep38174

**Published:** 2016-12-01

**Authors:** A. Perfetti, S. Greco, R. Cardani, B. Fossati, G. Cuomo, R. Valaperta, F. Ambrogi, A. Cortese, A. Botta, A. Mignarri, M. Santoro, C. Gaetano, E. Costa, M. T. Dotti, G. Silvestri, R. Massa, G. Meola, F. Martelli

**Affiliations:** 1 IRCCS Policlinico San Donato, San Donato Milanese, Milan, Italy; 2 Department of Clinical Sciences and Community Health, University of Milan, Milan, Italy; 3 C. Mondino National Neurological Institute, Pavia, Italy; 4 Department of Biomedicine and Prevention, Tor Vergata University of Rome, Rome, Italy; 5 Institute of Neurology, Tor Vergata University, Rome, Italy; 6 Department of Medical, Surgical and Neurological Sciences, University of Siena, Italy; 7 Fondazione Don Carlo Gnocchi Onlus, Milan, Italy; 8 Goethe University, Frankfurt am Main, Germany; 9 Institute of Neurology, Catholic University- L.go F, Vito- Rome, Italy; 10 Department of Biomedical Sciences for Health, University of Milan, Milan, Italy

## Abstract

Non-invasive and simple to measure biomarkers are still an unmet need for myotonic dystrophy type 1 (DM1). Indeed, muscle biopsies can be extremely informative, but their invasive nature limits their application. Extracellular microRNAs are emerging humoral biomarkers and preliminary studies identified a group of miRNAs that are deregulated in the plasma or serum of small groups of DM1 patients. Here we adopted very stringent selection and normalization criteria to validate or disprove these miRNAs in 103 DM1 patients and 111 matched controls. We confirmed that 8 miRNAs out of 12 were significantly deregulated in DM1 patients: miR-1, miR-27b, miR-133a, miR-133b, miR-206, miR-140-3p, miR-454 and miR-574. The levels of these miRNAs, alone or in combination, discriminated DM1 from controls significantly, and correlated with both skeletal muscle strength and creatine kinase values. Interestingly, miR-133b levels were significantly higher in DM1 female patients. Finally, the identified miRNAs were also deregulated in the plasma of a small group (n = 30) of DM2 patients. In conclusion, this study proposes that miRNAs might be useful as DM1 humoral biomarkers.

Myotonic Dystrophy (DM), is one of the most common inherited muscular dystrophy in adulthood among Caucasians^1^,^2^. It is characterized by two forms: myotonic dystrophy type 1 (DM1, OMIM #160900) also called Steinert’s disease, and myotonic dystrophy type 2 (DM2), known as proximal myotonic dystrophy (PROMM, OMIM#602668)^2^. DM1 is associated with pathological expansion of unstable (CTG)_n_ repeats in the 3′ untranslated region of the *DMPK* gene, while DM2 is due to expansion of (CCTG)_n_ repeats in the first intron of *ZNF9* gene^3^.

The typical patient assessment includes the diagnosis by genetic testing, flanked by electromyography, skeletal muscle histopathology and magnetic resonance^3^. Creatine kinase (CK) activity remains the only humoral marker measured in DM patients, but it lacks disease specificity^4^ since it is perturbed in most disorders involving skeletal muscle damage, as well as by intense physical activity^5^. Alternative splicing alterations in skeletal muscle tissue have been proposed as disease biomarker^6^; however, this requires an invasive procedure and harvesting several biopsies to monitor disease progression seems unfeasible.

MicroRNAs (miRNAs) are small noncoding RNAs that can regulate their target genes at post-transcriptional level^7^. Several studies indicate a fundamental role of both muscle specific (myomiRs) and non-muscle specific miRNAs in skeletal muscle differentiation and function^8^,^9^. Interestingly, miRNA dysregulation has been found in many skeletal muscle dystrophies^10^,^11^,^12^,^13^, including DM1 and DM2^14^,^15^,^16^,^17^. miRNAs are considered as good potential biomarkers, since miRNAs derived from various tissues and organs are easily detectable in both plasma and serum^18^,^19^. Most importantly, plasma/serum miRNA levels are altered in many diseases, including muscular dystrophies^11^,^20^,^21^,^22^. In particular, we and others^23^,^24^ have identified a group of miRNAs, including also myomiRs, that are deregulated in the plasma or serum of DM1 patients. These studies indicate that the levels of specific miRNAs correlate with loss of muscle strength and disease stage, suggesting a diagnostic and/or prognostic potential.

Although very promising, the significance of these studies is limited by the low numerosity of patients analyzed: 36 for the study of Perfetti *et al*.^23^ and 23 for the study of Koutsoulidou *et al*.^24^. Given the need of humoral biomarkers of prognostic value and also potentially useful as outcome measure for future therapeutic trials in DM1 patients, in this work we have analyzed the miRNAs deregulated in DM1 in a larger and independent patients group, to corroborate or disprove the existing data.

## Results

### Validation of deregulated plasma miRNAs in DM1

Platelet-free plasma samples were harvested from 103 DM1, and 111 age- and sex- matched control (CTR) patients, displaying no neuromuscular disorders. DM1 patients presented the expected hallmarks of myotonic dystrophy, summarized in Table 1: myotonia, cataract and loss of muscle strength^4^. Most of the patients were in stage 3 of the disease (MIRS score)^25^ and the pathological CTG expansions ranged from 65 to 1400. Of note, there was no overlap between the patients and controls analyzed in this study and the ones analyzed in our previous investigation^23^. Total RNA was extracted from plasma samples and miRNAs were measured by qPCR. For this validation study, we measured miR-133a, miR-193b, miR-191, miR-140-3p, miR-27b, miR-454, miR-574, miR-885-5p and miR-886-3p, previously described in Perfetti *et al*.^23^, and miR-1, miR-206 and miR-133b identified by Koutsoulidou and collaborators^24^. Normalization is a critical issue in all circulating miRNA studies^26^. Three normalizers were used, one exogenous (cel-miR-39) and two endogenous (miR-106a and miR-17-5p). Exogenous spike-ins, such as cel-miR-39, have been widely used for normalization purposes^11^,^26^,^27^,^28^ and we previously described the use of miR-106a and miR-17-5p as stable endogenous normalizers in DM1 patients^23^. Additionally, our previously published profiling study displayed no evidence of global changes of plasma miRNAs associated to DM1^23^.

The high number of DM1 patients and of miRNAs tested required several qPCR sessions. To make sure to have comparable results, we used a reference sample obtained by pooling a fraction of all control preparations. By measuring this reference sample in each qPCR session, we minimized technical variability throughout the study.

**Figure 1:**
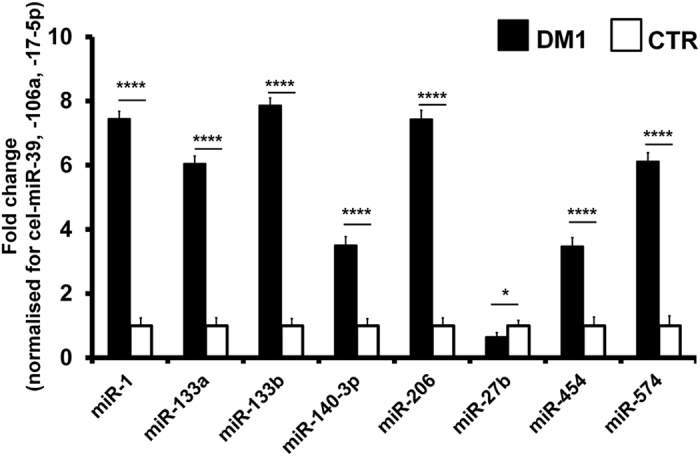
Validation of plasma miRNAs in DM1 patients. The bar graph represents mean values of the indicated miRNAs in plasma of DM1 patients compared to controls (CTR). Average values of cel-miR-39, miR106a and 17-5p were used for normalization (DM1 n = 103, CTR n = 111; ****p < 0.0001, *p < 0.05).

**Figure 2:**
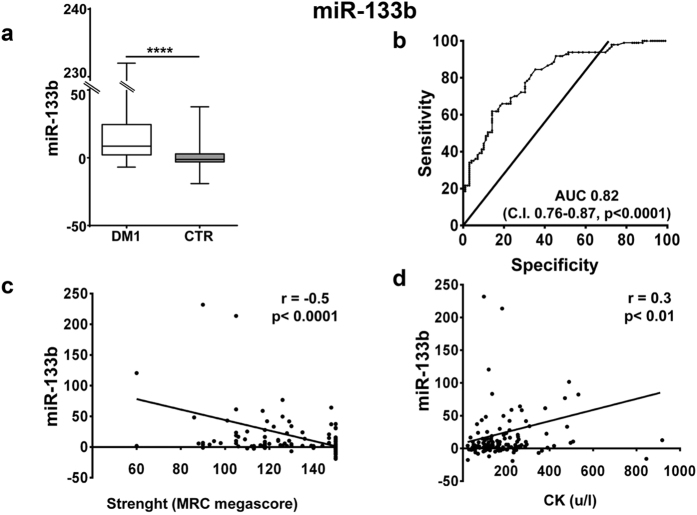
miR-133b validation as DM1 biomarker. (**a**) Box plot of miR-133b levels in the plasma of DM1 and controls (CTR; ****p < 0.0001). (**b**) ROC curve illustrating sensitivity and specificity of plasma miR-133b in discriminating healthy from diseased patients. (**c**) Spearman’s inverse correlation between miRNA-133b fold change compared to CTR and muscle strength measured by MRC megascore. (**d**) Spearman’s direct correlation between miRNA-133b fold change compared to CTR and CK plasma levels. For all panels, DM1 n = 103, CTR n = 111.

Given the importance of the normalization step and in order to maximize specificity, we considered as validated only those miRNAs that displayed significant differences after normalization with all of the following normalizers: (1) the average of cel-miR-39, miR-106a and miR-17-5p (Fig. 1); (2) exogenous cel-miR-39 only (Supplementary Table S1); (3) the average of the two endogenous miRNAs, miR-106a and miR-17-5p (Supplementary Table S1). Using these stringent criteria, we confirmed that 8 miRNAs out of 12 were significantly deregulated in DM1 patients. In particular, miR-1, -133a, -133b, -206, -140-3p, -454 and miR-574 were up-regulated, while miR-27b was the only down-modulated one. Notably, although lower standard errors and p values were generally observed upon normalization with three normalizers averaged, values obtained with all normalization methods were very similar, confirming the robustness of the data.

### DM1-deregulated miRNAs accurately identify DM1 patients

To test the differentiating value of the miRNAs validated in DM1 patients, we determined miRNA specific cutoff levels from Receiver–Operator Characteristic (ROC) curves (Supplementary Fig. S1). We found that miR-133b (Fig. 2) displayed the highest area under the curve (AUC = 0.82). Other myomiRs exhibited similar AUCs (from 0.79 to 0.81, Supplementary Fig S1a–d), while non-myogenic miRNAs displayed lower, but still significant values (from 0.58 to 0.71; Supplementary Fig. S1e–h).

**Figure 3:**
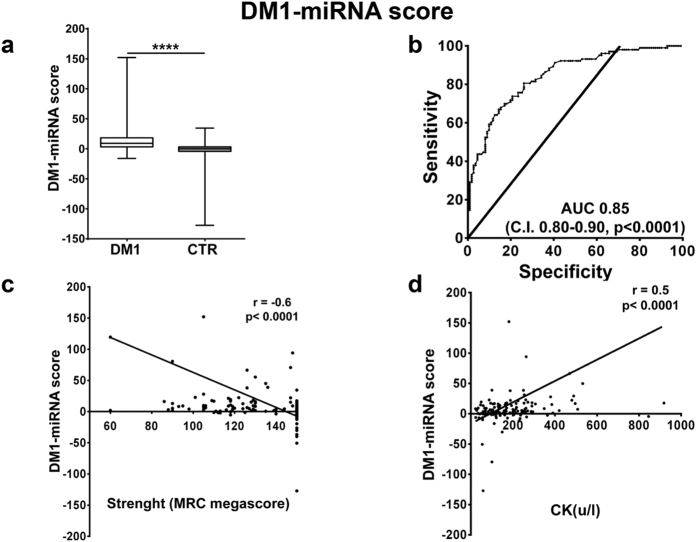
DM1-miRNA score validation as DM1 biomarker. The DM1-miRNA score was calculated averaging the fold changes of miR-1, -133a, -133b, -140-3p, -206, -27b, -454 and -574 in the plasma of DM1 and controls (DM1 n = 103, CTR n = 111). (**a**) Box plot of DM1-miRNA score levels in the plasma of DM1 and controls (CTR; ****p < 0.0001). (**b**) ROC curve illustrating sensitivity and specificity of the DM1-miRNA score in discriminating healthy from diseased patients. (**c**) Spearman’s inverse correlation between the DM1-miRNA score and muscle strength measured by MRC megascore. (**d**) Spearman’s direct correlation between the DM1-miRNA score and CK plasma levels.

**Figure 4:**
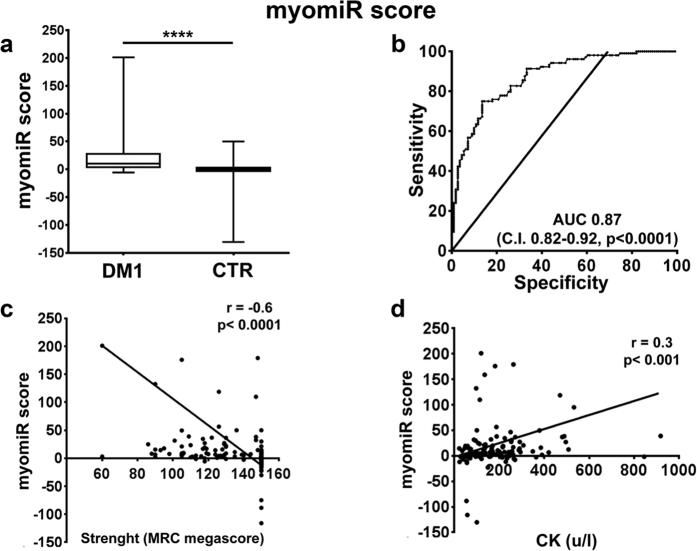
myomiR score validation as DM1 biomarker. The myomiR score was calculated averaging the fold changes of miR-1, -133a, -133b, and -206 in the plasma of DM1 and controls (DM1 n = 103, CTR n = 111). (**a**) Box plot of myomiR score levels in the plasma of DM1 and controls (CTR; ****p < 0.0001). (**b**) ROC curve illustrating sensitivity and specificity of the myomiR score in discriminating healthy from diseased patients. (**c**) Spearman’s inverse correlation between the myomiR score and muscle strength measured by MRC megascore. (**d**) Spearman’s direct correlation between the myomiR score and CK plasma levels.

**Figure 5:**
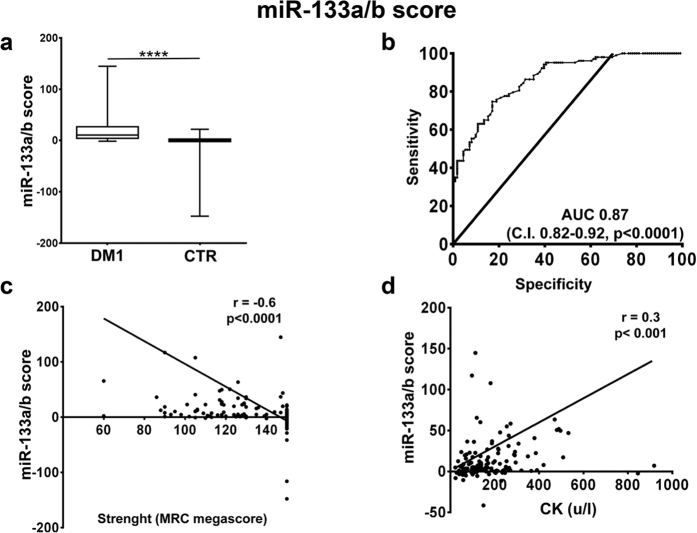
miR-133 a/b score validation as DM1 biomarker. The miR-133a/b score was calculated averaging the fold changes of miRNA-133a and -133b in the plasma of DM1 and controls (DM1 n = 103, CTR n = 111). (**a**) Box plot of miR-133a/b score levels in the plasma of DM1 and controls (CTR; ****p < 0.0001). (**b**) ROC curve illustrating sensitivity and specificity of the miR-133a/b score in discriminating healthy from diseased patients. (**c**) Spearman’s inverse correlation between the miR-133a/b score and muscle strength measured by MRC megascore. (**d**) Spearman’s direct correlation between the miR-133a/b score and CK plasma levels.

Next, we tested whether combining the values of different miRNAs allowed even better performances. To this aim, we calculated three scores, averaging the fold changes obtained for the relevant miRNA species: (1) a “DM1-miRNA score”^23^, averaging the fold changes obtained for all miRNAs (Fig. 3a); (2) a “myomiR score”, calculated averaging only the values of the myomiRs, i.e. miR-1,-133a, -133b and -206 (Fig. 4a); (3) a “miR-133a/b score”, averaging miR-133a and b, i.e. the top performing miRNAs of the two transcriptional units encompassing miR1-miR133a and miR-206-miR-133b^8^ (Fig. 5a). We found that all scores performed somewhat better than miR-133b alone, with AUCs of 0.85 for the DM1-miRNA score (Fig. 3b), 0.87 for the myomiR score (Fig. 4b) and 0.86 for the miR-133a/b score (Fig. 5b).

In particular, considering a cut-off value of 2.55, sensitivities of 81.6%, 82.3%, and 82.5% as well as specificities of 70.3%, 71.2% and 71.2% were observed for the DM1-miRNAs score, the myomiR score and the miR-133a/b score, respectively.

We also run a statistical analysis to identify outliers. Supplementary Figure S2 shows that, even after exclusion of the outliers, all differences remained statistically significant. Finally, we also found that, using different normalization procedures, similar AUC values were obtained for all scores, confirming the robustness of the data (Supplementary Table S2).

In conclusion, all three scores were useful in differentiating DM1 patients from controls.

### Correlation with clinically relevant parameters

We assessed the correlation of the identified scores with clinically relevant parameters. We found that, analyzing all subjects, miR-133b levels and all three scores displayed a weak but significant inverse correlation with muscle strength (MRC, Figures 2, 3, 4 and 5, panels c), and a weak direct correlation with CK values (Figures 2, 3, 4 and 5, panels d). One limitation of the latter correlation is constituted by the fact that exercise in the days before testing was not recorded.

**Figure 6:**
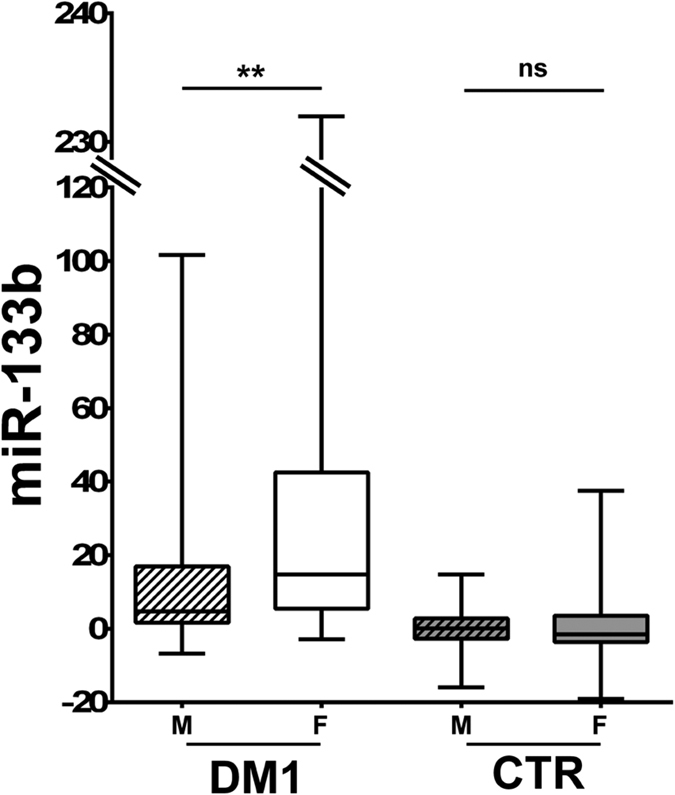
Higher miR-133b levels in female DM1 patients. The box plot shows miR-133b levels in male (M, striped boxes) and female (F, solid boxes) subjects (DM1 n = 103, CTR n = 111; **p < 0.01, ns = not significant).

Moreover, we also found that miR-133b levels were significantly higher in female DM1 patients compared to DM1 males, while no difference was observed in control subjects (Fig. 6). This difference was present with all three normalization procedures.

Next, to better investigate the relationship between clinically relevant variables in DM1 patients that were most affected by disease, i.e. with MIRS >2, a multiple linear regression was performed analyzing CK values, age of onset, number of triplet expansions and muscle strength. Statistically significant associations are reported in Table 2. We identified the association of muscle strength with miR-133b levels and all three scores. Moreover, CK values were associated to myomiR-score and miR-133a/b score.

We can conclude that all the identified scores display good combinations of sensitivity, specificity and correlation with clinically relevant features. Considering the minimization of the number of assays as an important parameter for the transferability to the clinical practice, also a miR-133a/b score obtained using only the two internal normalizers (Supplementary Table S4) displayed features very similar to these obtained using the three normalizers (Fig. 5).

### DM1-deregulated miRNAs are similarly modulated in DM2 patients

**Figure 7:**
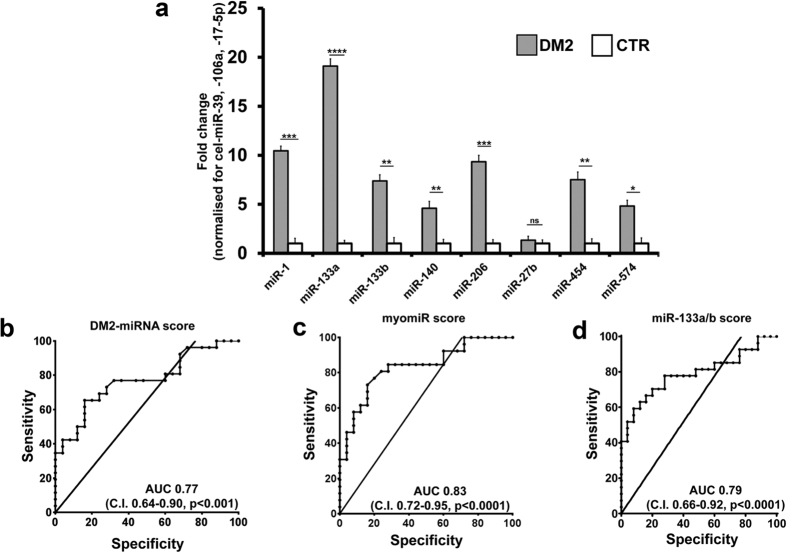
Validation of plasma miRNAs in DM2 patients. (**a**) The bar graph represents mean values of the indicated miRNAs in plasma of DM2 patients compared to controls (CTR). Average values of cel-miR-39, miR106a and 17-5p were used for normalization (****p < 0.0001, ***p < 0.001, **p < 0.01,*p < 0.05). ROC curves illustrating sensitivity and specificity of DM2-miRNA score (**b**), myomiR (**c**) and miR-133a/b score (**d**) in discriminating healthy from disease patients. (DM2 n = 30, CTR n = 30).

In order to assess whether the miRNA deregulations found in DM1 patients were also observed in DM2 patients, the plasma of 30 DM2 patients and 30 age and sex matched control subjects was assayed. Figure 7a shows that, with the exception of miR-27b, all the other miRNAs were significantly increased, further confirming the similarity of the two DM diseases.

Next we tested the differentiating value of the identified miRNAs in DM2 patients, calculating the same scores as for DM1, keeping out miR-27b. It was found that all three scores efficiently differentiated DM2 patients from controls (Fig. 7b,c and d).

## Discussion

DM1 is the most prevalent muscular dystrophy in adults; nevertheless there are very few humoral biomarkers that can facilitate the assessment of the patient clinical status^4^.

Plasma/serum miRNA hold a great potential as biomarkers in many diseases, including muscle dystrophies^13^. In spite of this, several hurdles exist for the successful application of circulating miRNAs as biomarkers. Indeed, one of the main issues in this kind of studies is that usually small patient groups are analyzed^11^,^21^, particularly in case of rare diseases, such as DM1. Considering the great variability existing from one patient to another and the absence of standardized procedures, this could lead to unreproducible tests not suitable for diagnostic routine. To overcome this hurdle, in this study we measured the same miRNAs previously found deregulated in plasma or serum of DM1 patients^23^,^24^ in a larger and independent group, in order to corroborate or disprove the existing data. Here we analyzed more than 200 subjects between DM1 patients and controls. Albeit still limited in size, this already represents a big step forward compared to similar studies performed in rare diseases^11^,^21^. Additionally, an accurate normalization method^23^ and stringent inclusion criteria were adopted. This approach allowed us to validate eight previously identified miRNAs, whereas four miRNAs were not confirmed, proving the importance of an accurate validation procedure. It is also worth noting that one of the RNA that were not confirmed, miR-886, is not even properly a miRNA, since it is identical to a fragment of Vault RNA 2 (VTRNA2)^29^, further supporting the superior performances of miRNAs as biomarkers. Finally, it is also worth noting that small studies also display low sensitivity. Indeed, several myomiR identified by Koutsoulidou, *et al*.^24^ escaped identification in our previous study^23^. In this study, we validated the myomiR miR-1, 133a,-133b and -206 as increased in the plasma of DM1 patients.

Both miR-133a/b and myomiR scores correlated, although weakly, with clinical parameters of muscle involvement, supporting the hypothesis that myomiR dysregulation in the plasma of DM1 patients is specifically related to mechanisms of muscle damage. Whether this was due to passive release from damaged myofibers, it is not known. However, unlike Duchenne muscle dystrophy, necrosis is not a DM1 disease hallmark and this increase might be due, at least in part, to active mechanisms. Interestingly, while myomiR levels are not modulated in DM1 skeletal muscle biopsies, their intracellular distribution is aberrant^15^, possibly leading also to increased extracellular release.

Also interesting is the fact that miR-133b elevation was particularly prominent in DM1 female patients. While the reason for this gender difference is unknown, it is not surprising since gender is emerging as an important factor influencing DM1 clinical profile^30^,^31^. Of note, it has been found that miR-133b stimulates ovarian estradiol synthesis^32^. Whether this is related to the endocrinological abnormalities observed in DM1 patients remains to be elucidated^1^,^2^.

Other non muscle-specific miRNAs, namely miR-140, -27b, -454 and -574 were also validated as deregulated in DM1 plasma. Indeed, since DM1 is a multisystemic disorder^3^,^33^, it is possible that the tissue of origin of these miRNAs might not be the skeletal muscle. According to the human miRNA tissue atlas (https://ccb-web.cs.uni-saarland.de/tissueatlas), they are expressed to different extent in multiple tissues, including brain, nerve, spinal cord, thyroid and epididymis. Thus, the plasma levels of these miRNAs may reflect the global clinical state of the patient, rather than that of a specific tissue. Notably, the new plasma signature has also been confirmed in a small group of DM2 patients. While confirmatory studies in larger patient groups are necessary, this finding suggests that similar mechanisms underpinning miRNA release in the blood might be shared by DM1 and 2. Standardized procedures and more refined quantitative measurements might also allow investigating possible differences between DM1 and DM2 in the extent of dysregulation of the identified miRNAs. As for DM1, miRNAs deregulated in the plasma of DM2 patients have not been reported to be deregulated in skeletal muscle biopsies^16^. Again, this is not surprising, since plasma miRNA levels are regulated by the combination of active and passive release of miRNAs in the bloodstream by all the tissues of the organism.

Three potentially useful scores were identified: the most simple is the miR133a/b that consists of only 2 miRNAs and that holds true even if only 2 normalizers are used, facilitating the potential transfer to the clinical practice. On the other side, the DM1-miRNA score, constituted by 8 miRNAs, is more complex, but displays better correlation with clinically relevant parameters. Moreover, it is not constituted only by myomiRs, that have been found to be deregulated in other dystrophies as well^11^,^20^,^21^,^22^. Thus, it holds the potential to be more DM-specific. However, this remains to be determined experimentally. Also important will be a longitudinal study, to ascertain how the identified scores change over time during DM1 disease evolution.

From a technical point of view, the identified miRNAs are little or not expressed in erythrocytes, minimizing the potentially distortive effect of hemolysis^34^. Very important for the potential transferability to the clinics will be the development of absolute quantification assays and the identification of reference values.

As a final remark, a humoral biomarker that may facilitate disease state monitoring of the patients using a minimally invasive procedure might be particularly useful considering perspective therapeutic options, as indicated by ongoing clinical trials (NCT02312011, NCT01406873).

## Methods

### Patient selection and plasma collection

All the experimental protocols were approved by the Institutional Ethics Committees of San Raffaele Hospital, Mondino Hospital and Policlinico Tor Vergata (protocol numbers miRNADM of 23.06.2015; 3584/13 of 07.25.2013; 145.14 of 10.23.2014) and were conducted according to the principles expressed in the Declaration of Helsinki, the institutional regulation and Italian laws and guidelines. All blood samples were taken after specific written informed consent. Clinical diagnosis of DM patients was based upon International Consortium for Myotonic Dystrophies guidelines^35^ and genetic analysis was carried out to confirm DM diagnosis, as previously described^36^. Stage of disease was determined using Muscular Impairment Rating Scale (MIRS)^25^. Five-point MRC scale (Medical Research Council) in the upper and lower limbs for a total maximum score of 150 (MRC megascore) was used to evaluate muscle strength^37^.

Blood samples were obtained from 103 DM1 patients, and 111 sex and age matched subjects displaying no overt sign of neuromuscular disorders (Table 1). Moreover, 30 DM2 patients (Supplementary Table S3) and 30 age and sex matched controls were also recruited (54.3 ± 2.3 years old, 18 males and 12 females). The control groups of DM1 and DM2 were only partially overlapping. EDTA-tubes were used for plasma preparation. Cell- and platelet-free plasma was prepared as previously described^23^. Potential hemolysis was tested measuring plasma absorbance at 570 and 600 nm wavelengths, where oxy-, deoxy- and carboxy-hemoglobin display similar absorbance^38^. All samples had hemoglobin <10 mg/ml with the exception of one (DM1–119) that was discarded.

### RNA isolation, miRNA measurement and miRNA scores

Total RNA was extracted as previously described using NucleoSpin miRNA Plasma columns (Macherey-Nagel)^23^. Preparations were spiked in with exogenous cel-miR-39 to assess the efficiency of RNA extraction. miRNAs were measured by qPCR using TaqMan microRNA assays, performed in duplicate according to the manufacturer instruction (Life Technologies). Raw Ct values are shown in Supplementary Table S4.

Relative expression was calculated using the comparative Ct method^39^. To calculate ∆Cts values, the average of 3 normalizers was used: spike in cel-miR-39 and 2 endogenous stable miRNAs, miR-106a and miR-17-5p, as previously described^23^. Next, to calculate ∆∆Ct values, DM1 and individual controls were all compared to a reference pool of CTR RNAs (control mix), measured in quadruplicate, ensuring comparable results throughout the study. Intra-assay variation for each miRNA is indicated in Supplementary Table S5.

For the score calculations, ∆∆Ct values were converted to linear values using the formula 2^(−∆∆Ct)^ for the up-regulations and the formula −2^(−∆∆Ct)^ for down-modulations^39^. Next “DM1-miRNAs score” was calculated averaging the fold changes obtained for all validated miRNAs. Since miR-27b was down-modulated in DM1, in this case the sign was inverted. For the “myomiR score” calculation, the fold changes obtained for miR-1, -133a, -133b and -206 were averaged. For the “miR-133a/b score” calculation, the fold changes obtained for miR-133a and b were averaged.

### Statistical analysis

Continuous variables are expressed as mean ± standard error (SE) unless indicated differently. The box plots represent data divided in quartiles. For group-wise comparisons, Mann–Whitney U test was used. The ability to discriminate between the DM1 and control groups was determined by the receiver operating characteristic curve, and the area under the curve was calculated. Spearman rank correlation was used to compare miRNA levels with clinical characteristics. For multiple linear regression, absolute ∆∆Ct were used to study the association between all the scores and clinical characteristics. The normality of the scores was assessed through histogram visualization. Outliers were identified by Tukey’s test. Bonferroni’s method was used to correct for multiple testing. All tests were performed 2-sided and a p ≤ 0.05 was considered as statistically significant. For statistical analysis GraphPad Prism v. 7.0 (GraphPad Software Inc.) and R^40^ software were used.

## Additional Information

**How to cite this article**: Perfetti, A. *et al*. Validation of plasma microRNAs as biomarkers for myotonic dystrophy type 1. *Sci. Rep.* **6**, 38174; doi: 10.1038/srep38174 (2016).

**Publisher's note:** Springer Nature remains neutral with regard to jurisdictional claims in published maps and institutional affiliations.

## Supplementary Material

Supplementary Information

Supplementary Table S1

Supplementary Table S2

Supplementary Table S4

## Figures and Tables

**Table 1:** Clinical characteristics of DM1 and control patients.

Clinical Characteristics	DM1, n = 103	CTR, n = 111
Age at sampling (average ± se)	44.1 ± 1.3	43.0 ± 1.0
Age of onset (average ± se)	25.9 ± 1.3	NR
Sex (male/female)	58/45	51/60
MRC megascore (average ± se)	118.6 ± 1.9	150.0 ± 0.0
Myotonia (% of patients)	87	0
Glucose (normal values: 70–110 mg/dl)	88.7 ± 1.2	90.3 ± 1.3
Cholesterol (normal values: <200 mg/dl)	212.0 ± 6.7	204.0 ± 6.0
CK (normal values: male < 190 mg/dl, female < 125 mg/dl)	Male: 280 ± 3.5	Male: 128.8 ± 27.2
	Female: 224.6 ± 22.7	Female: 124.5 ± 24.1
Arrhythmia (% of patients)	38	0
Cataract (% of patients)	56	0
ECG-QRS duration (normal values: 60–110 ms)	103.5 ± 4.3	NA
Number of CTG repeats (range)	484.0 ± 27.6 (65–1400)	NA
Stage of disease (range 1–5) (% of patients at each stage)	Stage 1: 5	NR
	Stage 2: 29	
	Stage 3: 34	
	Stage 4: 28	
	Stage 5: 3	

NR: not relevant, NA: not available.

**Table 2:** Multiple regression analysis, DM1 patients with stage of disease higher than 2.

SCORE	PARAMETERS	COEFFICIENT	STD ERROR	p-value
DM1-miRNA	ΔMRC^a^	0.036	0.013	0.011
myomiR	ΔMRC	0.042	0.015	0.007
	CK	0.004	0.002	0.033
miR133a/b	ΔMRC	0.038	0.014	0.011
miR-133b	ΔMRC	0.059	0.019	0.005
	CK	0.005	0.002	0.035

^a^ΔMRC is the difference between MRC megascore reference value (150), and MRC of each patient.
