# Erratum: Validation of plasma microRNAs as biomarkers for myotonic dystrophy type 1

**DOI:** 10.1038/srep43074

**Published:** 2017-02-22

**Authors:** A. Perfetti, S. Greco, R. Cardani, B. Fossati, G. Cuomo, R. Valaperta, F. Ambrogi, A. Cortese, A. Botta, A. Mignarri, M. Santoro, C. Gaetano, E. Costa, M. T. Dotti, G. Silvestri, R. Massa, G. Meola, F. Martelli

Scientific Reports 6 Article number: 3817410.1038/srep38174; published online: 12 01 2016; updated on: 02 22 2017

This Article contains errors in the Affiliations of M. Santoro and A. Mignarri. The correct affiliations for these authors are listed below:

M. Santoro

Fondazione Don Carlo Gnocchi Onlus, Milan, Italy.

A. Mignarri

Department of Medical, Surgical and Neurological Sciences, University of Siena, Italy.

