# Supplementary material for: Validation of plasma microRNAs as biomarkers for myotonic dystrophy type 1
Source: Sci Rep. 2016 Dec 1;6:38174. doi: 10.1038/srep38174 (PMC5131283; doi:10.1038/srep38174)
Supplement: Supplementary Information [file srep38174-s1.pdf]

Supplementary informations

Validation of plasma microRNAs as biomarkers for myotonic dystrophy type 1.

Perfetti A., Greco S., Cardani R., Fossati B., Cuomo G., Valaperta R., Ambrogi F., Cortese A., Botta A., Mignarri A., Santoro M., Gaetano C., Costa E., Dotti M.T., Silvestri G., Massa R., Meola G., Martelli F.

Supplementary figure S1

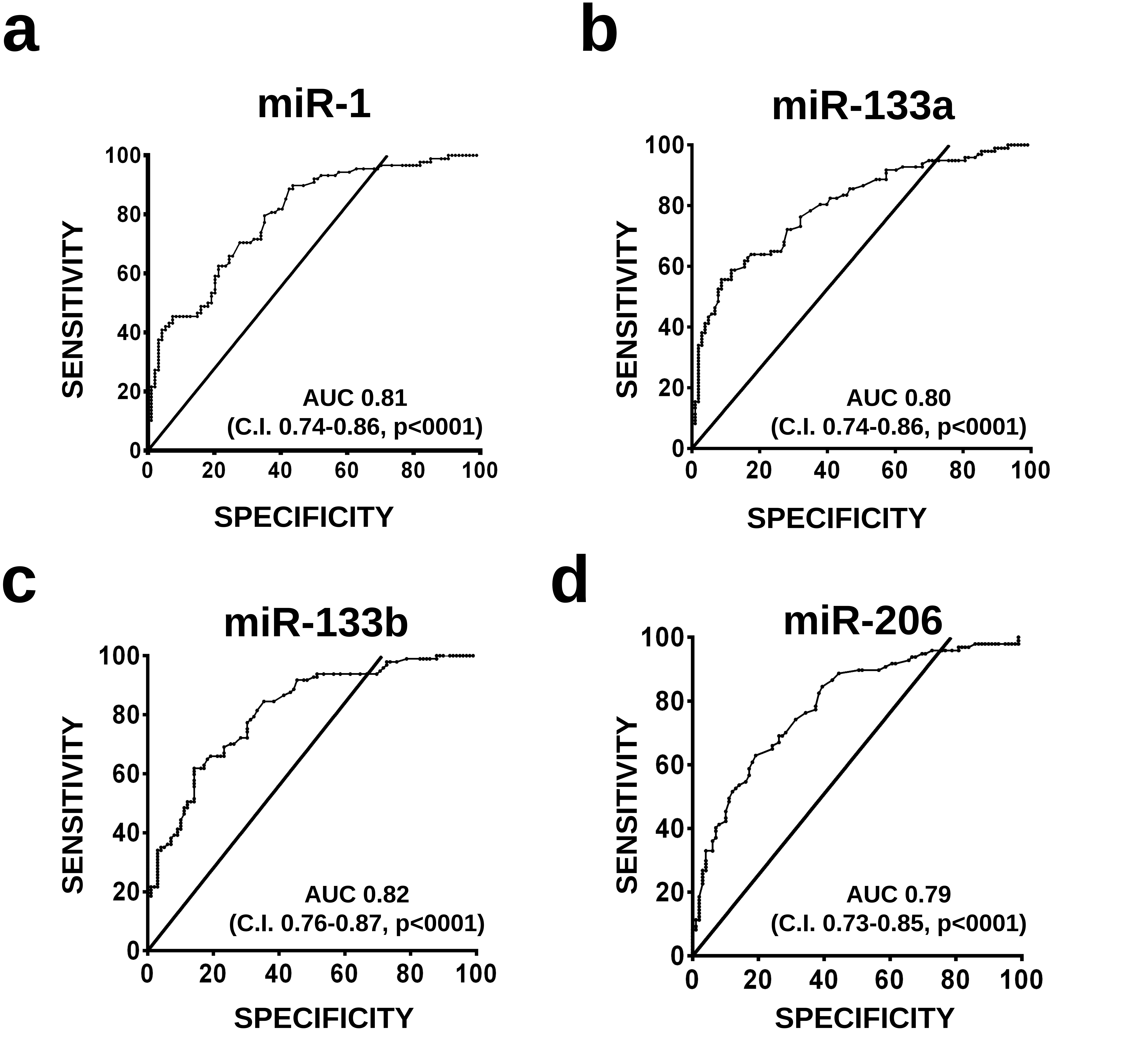

Supplementary figure S1

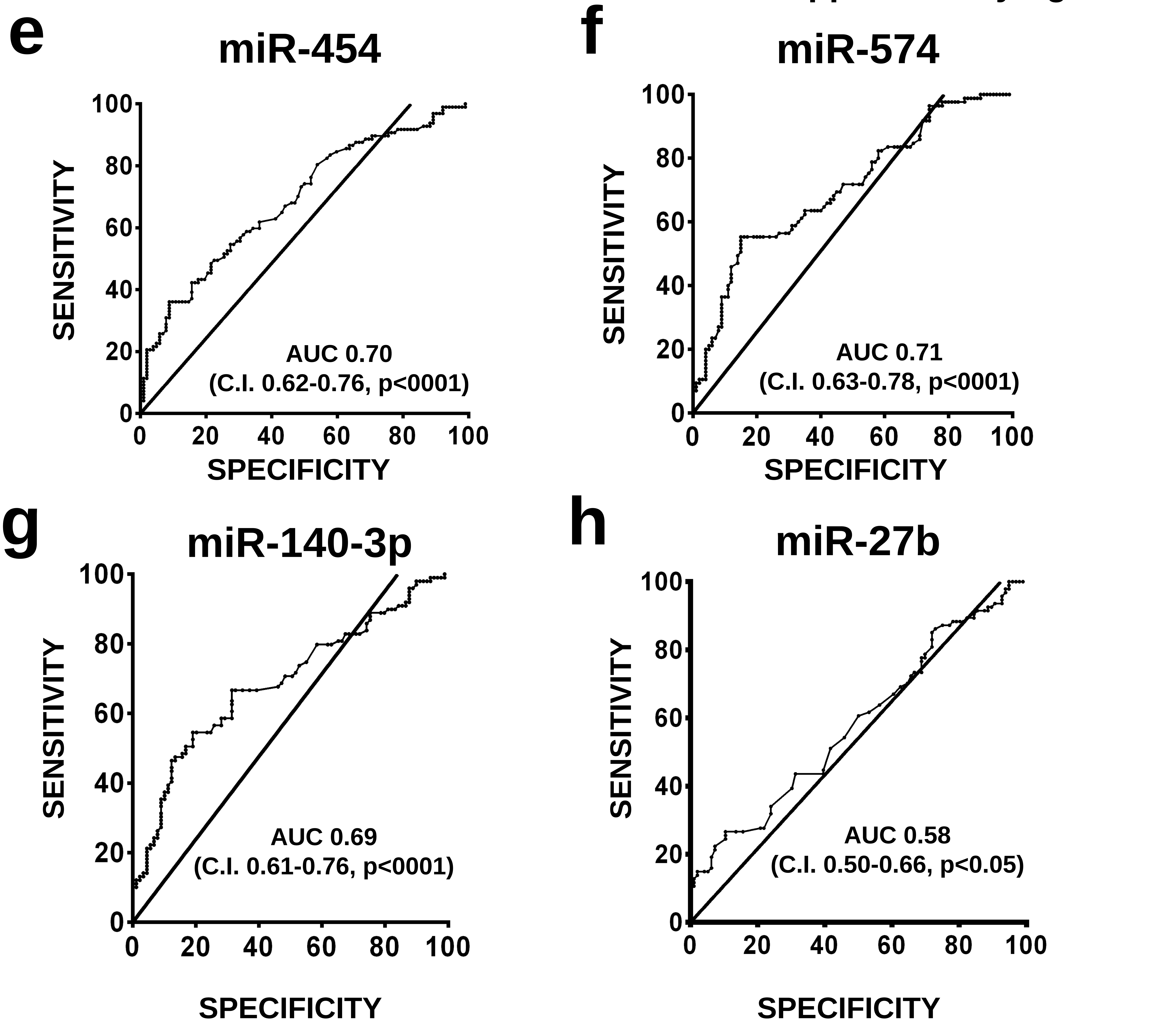

Supplementary figure S1. ROC curves of each validated miRNA. Area under the curve (AUC) is indicated for each modulated miRNA (DM1 n=103, CTR n=111).

Supplementary figure S2

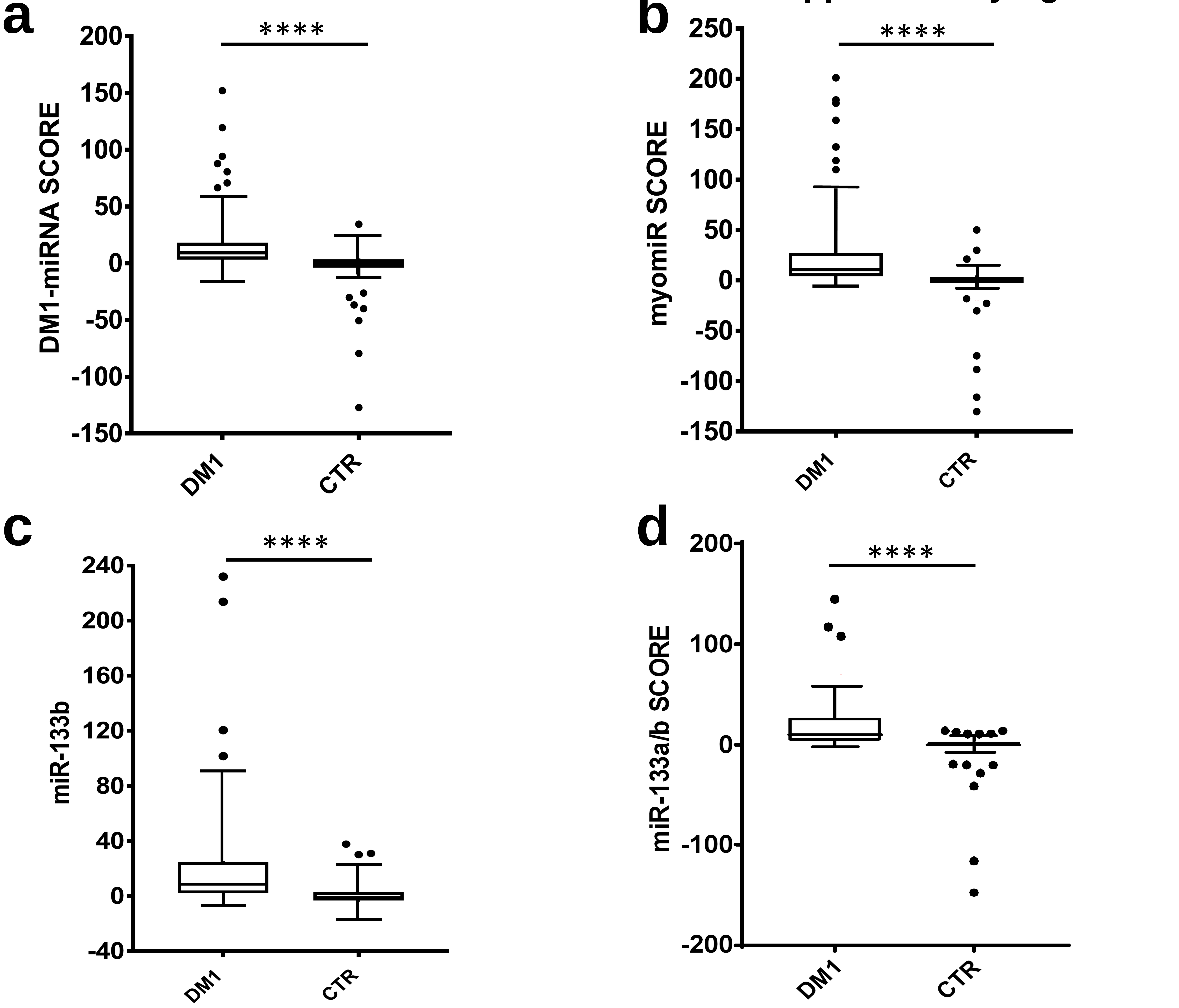

Supplementary figure S2. **Outliers identification by Tukey's test** . DM1-miRNA score (a), myomiR score (b), miR-133b (c) and miR-133a/b score (c) are represented after exclusion of outlier samples (individual dots) identified by Tukey's test. After outliers exclusion all differences were statistically significant (****p<0.0001).

Supplementary table S3

CLINICAL CHARACTERISTICS	DM2, n=30
**Age at sampling (average ± se)**	54.7 ± 2.7
**Age of onset (average ± se)**	41.5 ± 2.4
**Sex (male/female)**	13/17
**MRC megascore**	143.5 ± 1.4
**Myotonia (%)**	54
**Glucose (70-110 mg/dl)**	112.3 ± 13.6
**Cholesterol (<200 mg/dl)**	230.1± 9.6
**CK**	Male: 313.9±86.6
**(male<190 mg/dl, female <125 mg/dl)**	Female: 353.6.6 ±95.0
**Arrhythmia (%)**	12
**Cataract (%)**	32
**ECG-QRS duration (60-110 ms)**	99.1±4.5

Supplementary table S5

Intra-assay coefficients of variability (CV)

	AVERAGE CV %
**miR-1**	1,5
**miR-133a**	1,1
**miR-133b**	1,2
**miR-140**	1,0
**miR-206**	1,2
**miR-27b**	1,9
**miR-454**	1,2
**miR-574**	1,3
**miR-39**	1,7
**miR-106a**	2,0
**mi-17-5p**	1,9

DM1, n=103; CTR, n=111
